# On Erysipelas of the Head and Face

**Published:** 1848-07

**Authors:** C. S. Clary

**Affiliations:** Brandenburg, Kentucky


### *On Erysipelas of the Head and Face.* By C. S. Clary, M. D., of Brandenburg, Kentucky.

*Mr. Editor,*—I propose, in the following observations, to state a few facts in relation to a very interesting disease, which has prevailed to a considerable extent in this vicinity since the first of February last. I allude to erysipelas of the head and face, sometimes called by the practitioners of the south-western and western states, epidemic and contagious erysipelas, (one or both.) In common parlance the disease is known in this State, Indiana, Illinois, and Missouri, by the name of *black tongue.* In some localities the mortality is frightful, and our art impotent for its relief. At and near New Madrid, on the Mississippi, just below the mouth of the Ohio, I am informed that fully three-fifths of the cases result in death, and at Padducah in the lower part of this State, the mortality is nearly as great. But I will confine myself to my own neighbourhood, and as I write from memory and very imperfect notes, let me hope to be excused for lack of method.

About the first of February last, Miss E. R. landed at this place from a little town a few miles below on the river, to which place she had been on a visit to her brother, who was sick with erysipelas of the head and face. He died. Miss E. R. had a severe attack of the disease, but recovered. Before she was entirely well, Mrs. A., her hostess and principal nurse, was attacked with the same disease: also Mrs. R. a relative of the first named lady, who had visited her twice from the country. In her family, (Mrs. R.'s) which was very large, consisting of both white and black, there was no other case. During the illness of Mrs. A., she was visited by a servant girl belonging to Mr. C., and she was soon after taken sick and died. There was no other case in this family; but a week after the death of the girl just alluded to, a neighbouring black woman belonging to Mr. B. was engaged by the family to wash the clothes and cleanse the room of the deceased. About five or six days afterwards, this woman was attacked with erysipelas, during the progress of which she had an abortion, but recovered. She has since died of inflammation of the stomach and bowels. The owner of this woman, Mr. B., has also had erysipelas of one side of the neck and shoulder, which resulted in deep-seated suppuration, and the discharge of a large quantity of pus. In this family there was no other case, though Mr. B.'s three servants and four children were equally liable to be attacked, especially Mrs. B. and one servant, who were greatly fatigued and nearly exhausted by constant nursing. During the illness of Mrs. A., she was visited by a black woman, the property of Mr. L., living five miles in the country. This woman soon after had a severe attack of the disease, and communicated (?) it to all her children, eight in number. Her husband escaped it, though constantly at her bed-side, and he was the only member of the family black or white who *did* escape. The master and mistress (both old) and a young lady, a relation, all had it, and the two former died. Mrs. S., in town, had seen no one labouring under the disease, and as she was very timid, was careful not to go near a case of the kind : yet she had it, and though she was surrounded by a large family of children, who were *all the time of her illness* in the same room, still her own was the only case in the family. She was visited during her illness by a number of persons, and of all these no one took the disease, except the wife and daughter of Mr. D., but the attack in these two cases was six weeks after the exposure—a long period of incubation!

I have now alluded, in very general terms, to all the well marked cases of this disease that have come under my observation, as well as some others that I did not see at all, or until they were convalescent. The question, is erysipelas a *contagious* disease, in the strict and etiological acceptation of the term, remains to be answered.

Elliotson and several other English writers, believe erysipelas to be really contagious. Watson says, " it sometimes arises from contagion, but much more frequently from other causes," (I quote from memory) and with him undoubtedly rests the weight of authority. Strictly contagious diseases arise from their own *specific* causes, and no other. In this sense of the term, if I am asked whether I believe erysipelas to be a contagious disease, I unhesitatingly answer no, and that arguments are by no means conclusive, which go to prove that the disease is propagated in this way, even to a limited extent. The facts detailed above, do certainly *appear* strongly to favour the notion of contagion, but if the disease is "as contagious as small pox," why were there no other cases in the family of Mrs. A. besides her own and that of Mrs. R.? There were many others equally exposed. Why was Mrs. R.'s case the only one in her family? Why was the servant girl the only case in the family of Mrs. C.? and why again were the circumstances the same in the family of Mrs. S.? I leave the advocates of contagion to answer these questions. But it may be asked "how will you account for the cases of Miss E. R., Mrs. A., Mrs. R., and the family of Mr. L.?" I answer, that when the season of the year, the state of the weather, and the constitution of the atmosphere bear a certain relation to each other and to the human system, they induce what is called a *predisposition* to erysipelas or some other disease, as the case may be; the epidemic itself depending upon the season of the year in combination with other causes, which may be either local and temporary, or general, and of considerable duration. There must be such a thing as an "epidemic constitution of the atmosphere," else why is it that we observe the same disease prevailing at different times as an epidemic under such apparently different circumstances. The great Sydenham observed these things and made the proper inference. His theory of epidemics (if I may call it a theory) may be thought a vague one, and yet it is perhaps the best we have ever had.

A few years ago I was called to see a young man who had just returned to his father's house, after a visit of some days at a distance; he had fever, pain in the chest, cough, rusty expectoration, hurried respiration, and a red and sometimes livid spot on his cheek, together with all the physical *signs* of pneumonia, and such I pronounced it. In a few days, three young ladies of the same family (his sisters) were taken with the same disease, and in all four the symptoms were so exactly similar, that there was in fact, no appreciable difference, save what was occasioned by the difference of sex. From that time, during a period of six weeks, there were a goodly number of cases of pneumonia, principally among those who visited the four first cases. The *community* believed the disease contagious, but I am certain it was not, and every intelligent physician will agree with me. There was nothing typhoid about the disease; there was more or less hepatic derangement in all the cases, and all that can reasonably be said is, that a combination of causes favoured the prevalence of the malady as an epidemic at that particular time. Exposure, anxiety and watching, probably acted as the exciting causes. Do not these last examples furnish as strong arguments in favour of contagion as the cases of erysipelas of which I have been speaking? An individual labouring under a bilious remitting fever, may land from a steamboat at the hotel of Mrs. A., Mrs. A. may have an attack of the same, and of the persons who visit these two, a few may go to bed with the like disorder. The season of the year may be October, at which time a very large proportion of cases in this region are of this character, yet where is the medical man who would pronounce these examples of contagion?

Such instances have often occurred, and will most assuredly occur again and again. In seasons when influenza is prevailing as an epidemic, we frequently see one case in a family, and very soon every member, without one solitary exception, labouring under the same disease. Yet who believes influenza to be contagious? I have seen, in this county, in more instances than one, intermitting fever commence in a family, and soon every member of that household would be prostrated by the same malady. So, too, it is not unfrequently the case that dysentery will attack every member of a family when it once begins. Yet who pretends that either of these last named diseases is contagious? The truth is, erysipelas would have prevailed here even though Miss E. R. had not landed in town at all. There were just at that time, and before, several cases in and near a little village twelve miles east of this place, and Dr. C., who attended them, informed me that, after the most patient investigation, he could detect not the slightest evidence of contagion as a cause. Dr. M., fifteen miles south of me, also attended a number of cases and expressed the same opinion. Both of these gentlemen reside in this county, and are men of more than ordinary medical intelligence. The cases which came under their observation occurred about the same time with those noticed by myself. An unusual panic has prevailed here upon the supposition that the disease is remarkably CATCHING; fear has overpowered the better feelings of the heart, and in some instances the sick have greatly suffered for want of that attention and those kindly offices for which, in the country, they are always so entirely dependent upon the "goodness and mercy" of their neighbours. In the family of L——, all the negroes were housed in one room, not more than twelve feet square, more like the hold of a slave-ship than anything else to which I can compare it. When it is known that this room contained four beds filled with the sick—that they had no nurses out of the family—that the cases generally succeeded each other after intervals of several days—that those most fatigued and exhausted as nurses were the first taken down, (after the first case)—in short, when all the circumstances I have named are taken into consideration, surely we stand in small need of invoking the powers of contagion in order to enable us to assign a proper cause for erysipelas.

At the very time Miss E. R. landed here, the persons who *took* the disease from her were complaining of *sore throat*, and in fact had the premonitory symptoms of BLACK TONGUE. So universal has been the prevalence of this symptom, that I presume nine out of every ten of the population of our county have suffered from it more or less; this is another illustration of the law of epidemics.

I will now glance briefly at the treatment, premising, however, that of fourteen cases treated in part by myself three died. Of those treated exclusively by myself I lost but one. This case was the servant girl of Mr. C., alluded to above. I made a post-mortem examination, and found her lungs, especially the left, filled with large deposits of tuberculous matter, and the mesenteric glands so much enlarged and run together as to form of the whole only three or four large masses. She had been previously treated for scrofula. I mentioned before that the woman belonging to Mr. B. recovered of the erysipelas, but died subsequently of inflammation of the bowels. In the cases of the old gentleman and lady, Mr. and Mrs. L., I was compelled to abandon them six or seven days before they died, as they saw fit to send for a Thomsonian, but I

was sent for to see them again when they were in *articulo mortis.* One of them, I think, would have died under any medical treatment, unless the family had been better supplied with nurses.

When erysipelas is prevailing as an epidemic, if I am called to a patient who has had a chill, or an unnatural *chilly sensation,* and complains of much sickness and oppression of the stomach, sore throat, and a burning pain in any part of the body, I consider it as almost certain that I have a case of the disease. I look into the mouth, and if I see in the back part, over the palate and tonsils, &c., a red *fiery rash,* which seems to be spreading, in short, an erythematous inflammation, I then am quite sure that have a case that will eventually prove to be erysipelas of the head and face. The fauces, upper part of the œsophagus and the larynx, are generally loaded with a very tenacious mucus. In this stage of the case I give an emetic that will evacuate the stomach thoroughly, unless very evidently contraindicated. Next I open the bowels with rhubarb and soda, or any of the neutral saline purgatives in combination with ten or fifteen grains of the sup. carb. soda. Then I administer three grains of sulphate of quinine every hour, until the patient becomes sensible of a ringing in the ears, or until twenty-four grains are given, if an adult, and less according to the age of the individual. If the pain and swelling of the throat increase, I repeat the emetic and quinine the second day and also the third. I believe this course of emetics will, in a large majority of cases, prevent, to a very considerable extent, the otherwise enormous swelling of the head, face and throat. The emesis produces a concussion of the whole system, and seems, as it were, to dissipate and resolve the disease. If the disorder progresses, however, and the swelling is allowed to go on, there is nearly always more or less delirium. In these cases I would, if practicable, *bleed from the nose* by scarifying the Schneiderian membrane, which will generally be followed by decided improvement in the symptoms. The parts in the vicinity of the fauces should be kept clear of the tenacious mucus with which they are constantly loaded by gargling with Cayenne pepper. I have derived advantage by administering every hour a teaspoonful of the following mixture in a little warm water.

℞ Pulv. Capsic. ʒi.

Sodii Chlorid., ʒii.

Aq. Ferv., ℥vi. M.

We must guard against debility, which is so great in all the cases of considerable swelling as to constitute the principal cause of alarm to the physician. The pulse frequently numbers 140 to 150 for forty-eight hours. Here wine and carbonate of ammonia should be administered freely, until we increase its volume and lessen its frequency. In one case I gave a quart of wine during one night. As far as my experience goes, *general* bleeding is inadmissible, though I have no doubt that cases do frequently occur where it might and ought to be resorted to. The judicious practitioner will have an eye to the character of the prevailing epidemic, and then be guided by the circumstances of each particular case. With respect to the local treatment, the best probably consists in anointing the swollen surface twice or thrice daily with fresh hog's lard, and applying poultices made by thickening a decoction of hops with ground flaxseed, wheat bran, or corn meal. As to surrounding the part, and painting it with nitrate of silver, iodine, &c., for the purpose of preventing the progress of the swelling, I have no faith in it. In many cases where these appliances have been resorted to, and the advance of the tumefaction has *appeared* to cease, the physician has frequently been able to impose upon the friends of the patient, (especially if ignorant,) and induce them to look upon a *coincidence* as a *sequence.* The truth is, that under exactly the same treatment, in a given number of cases, there will be every imaginable difference as to the extent of the tumefaction. In one case it will be confined to the throat; in another it will extend to the ears and top of the under lip; in a third the effusion into the subcutaneous tissue will be so great as to close both eyes; in a fourth the disease will run over the whole face and scalp, occasioning the most hideous deformity; and in a fifth, as it leaves the head, it will travel over almost the whole body, terminating at the hands and feet. When it becomes necessary to administer a *placebo,* we may safely paint the part with a feather dipped in a solution of iodine and iodide of potassium, fifteen grains of the former and twenty of the latter to the ounce of rain-water, and repeat three or four times daily.

I think I have now given an *outline* of the best treatment for this formidable disease. As to the question of contagion, the influence of particular localities, the season of the year, and the modifying effects of *miasmata*, many facts will doubtless be collected which will add no little to our knowledge of erysipelas. *Magna est veritas et prevalebit* is a maxim which has stood the test of time, and yet its verification is sometimes most discouragingly slow.

*Brandenburg*, June, 1848.

